# Energy Efficient Cooperation in Underlay RFID Cognitive Networks for a Water Smart Home

**DOI:** 10.3390/s141018353

**Published:** 2014-09-30

**Authors:** Adnan Nasir, Syed Imtiaz Hussain, Boon-Hee Soong, Khalid Qaraqe

**Affiliations:** 1 Electrical and Computer Engineering Department, Texas A&M University at Qatar, Doha 23874, Qatar; E-Mails: syed.hussain@qatar.tamu.edu; khalid.qaraqe@qatar.tamu.edu; 2 School of Electrical and Electronic Engineering, Nanyang Technological University, 639798, Singapore; E-Mail: ebhsoong@ntu.edu.sg

**Keywords:** leak detection, selective cooperation, cognitive networks, RFID, underlay networks and water monitoring, smart homes

## Abstract

Shrinking water resources all over the world and increasing costs of water consumption have prompted water users and distribution companies to come up with water conserving strategies. We have proposed an energy-efficient smart water monitoring application in [1], using low power RFIDs. In the home environment, there exist many primary interferences within a room, such as cell-phones, Bluetooth devices, TV signals, cordless phones and WiFi devices. In order to reduce the interference from our proposed RFID network for these primary devices, we have proposed a cooperating underlay RFID cognitive network for our smart application on water. These underlay RFIDs should strictly adhere to the interference thresholds to work in parallel with the primary wireless devices [2]. This work is an extension of our previous ventures proposed in [2,3], and we enhanced the previous efforts by introducing a new system model and RFIDs. Our proposed scheme is mutually energy efficient and maximizes the signal-to-noise ratio (SNR) for the RFID link, while keeping the interference levels for the primary network below a certain threshold. A closed form expression for the probability density function (pdf) of the SNR at the destination reader/writer and outage probability are derived. Analytical results are verified through simulations. It is also shown that in comparison to non-cognitive selective cooperation, this scheme performs better in the low SNR region for cognitive networks. Moreover, the hidden Markov model’s (HMM) multi-level variant hierarchical hidden Markov model (HHMM) approach is used for pattern recognition and event detection for the data received for this system [4]. Using this model, a feedback and decision algorithm is also developed. This approach has been applied to simulated water pressure data from RFID motes, which were embedded in metallic water pipes.

## 1. Introduction

The pressure provided by pumps and roof tanks is needed to deliver water to consumers. From this pressure information, we can determine the water flow, which is the direct measure of the water usage. The pressure change gives unique signatures for different taping points, like the kitchen sink, the washing machine, the wash basin and showers. Furthermore, the change in pressure information in a single section of the pipe shows a possible leak or seepage. In our previous work, using PipeSense [1], an RFID-based in-pipe monitoring system, we have determined the feasibility of developing a monitoring system to measure the quality of water. The shrinking water resources all around the world have made it absolutely necessary for us to conserve water; this fact has also made water quite expensive. Usually for us, there is no way to know how much water has already been consumed, nor the cost of this consumption, until the bill arrives at the end of the month. Moreover, the bill is not easy to interpret, and it is difficult to extract information about consumption during a particular period of time. Secondly, there are unpredictable leaks or seepage occurs in the water pipes, accumulating the costs of the utility, the details of which remain unobserved; leaks alone are the reason for considerable loss in terms of money and resources per year. Repairing these leaks also incurs a cost and leads to the wasting of time and effort. We lack a wireless automatic system that can measure water usage in real time, monitor events, such as tap opening and closing, daily consumption patterns and predict possible leaks and seepage developing in the pipe network. For this system, we need to come up with methods and algorithms to localize the leaks and seepage in smart homes.

RFIDs integrated with pressure sensors are embedded in the pipe infrastructure. They collect pressure information and send it along with their IDs to the reader/writer destination node. From the available pressure data from the sensors, the determined usage patterns, tap events and their patterns assist the real-time control of the home water system. The information from the sensors is then run by the algorithms on the cyber system to render decisions in order to support the hardware controllers responsible for managing the water distribution parameters. Several methods have been used in the setting of multi-agent systems; however, we have chosen the hierarchical hidden Markov model (HHMM), which is a famous tool for pattern recognition [4,5]. The framework described in [4] is an integrated set of two separate four-tier frameworks; here, two HMMs were discussed for determining the *CO*_2_ and *CO* levels from the vehicle’s exhaust. These two HMMs were separate and are not dependent on each other, as the data is coming from two different sensors; while the HHMMs proposed in our current proposal are hierarchical in nature, due to the fact that the consumption patterns, tap and seepage event data are interdependent or, in some other way, coming from the same sensors. Normally, HMMs are used as a speech recognition tool world over. Due to the similarities of the sensor data that we received from the pipes to speech signals, we adopted the HHMM to solve the problem presented in the proposed solution. We can also make the smart home water system consumer friendly by making the data visualization easy to interpret, along the same lines as [6]. The integrated RFID motes collect and send the data to the reader/writer mote. We propose an event detection and prediction scheme based on HHMM, which is a hierarchical variant of HMM [7]. Our approach would be to detect the differences by statistically comparing the observed pattern with that predicted by a model to discover events of interest while minimizing the delays and false alarms. A preliminary version of this research appeared in [3]. The framework described in [3] is utilized for a human-centric in-pipe water distribution monitoring system (WDS) with the goal of determining the patterns and probabilities of future water demand, water quality and contamination spread using HMM; while our proposed work uses HHMM instead of HMM to determine the consumption pattern, tap events and seepage events in the context of a water smart home.

The term “smart home” has been used to introduce the notion of networking devices and equipment that distribute information and commands among the networked devices in the home via wired and wireless communication [8]. A smart home can accommodate a number of information gadgets, home appliances and other Internet-based applications, which can communicate with each other, forming a ubiquitous home network system [9]. This presents the idea of having a server-based home gateway system, which becomes the brain of the smart home, which will surely make life easier for the common household. However, there exist several other wireless devices simultaneously operating under the same roof. These two systems are bound to interfere with each other, resulting in higher energy consumption due to handshaking protocols. Hence, cognitive underlay networks come to the rescue in this scenario.

The available wireless spectrum has become a scarce resource, due to the rising demand for high data rate wireless services. Due to this scarcity, emergent communication systems are required to exploit the unused licensed spectrum in an opportunistic fashion [10]. It is proposed in [11] that in the case that the licensed or primary user of the spectrum is inactive, any cognitive or secondary users can use this available spectrum. When the primary user becomes active, the secondary user must switch off its communication and look for another spectrum hole. This technique is generally referred to as the interweave approach and involves spectrum sensing and detection. Generally, the overlay method simultaneously allows both the primary and secondary users to approach the spectrum; however, the interference for the primary user is subjugated by the secondary user through advanced signal processing. Another approach, called the underlay approach, allows the sharing of the spectrum by both primary and secondary users simultaneously. In this approach, the secondary user has to satisfy strict interference constraints, and its transmission power should be below a certain threshold all of the time [11]. Underlay cognitive networks use very limited transmission powers and, hence, make the system energy efficient. A subset of these RFID motes were allowed to send the data, which increases the received SNR ratios and will be useful in decision making. In our monitoring application, for better parameter monitoring, we will need to accumulate the maximum number of nodes satisfying the threshold requirements to increase the overall received SNR.

The work by Akbar *et al.* [12] presents the use of hidden Markov models (HMMs) to model and predict the spectrum occupancy of licensed radio bands. The proposed HMM by Akbar *et al.*, dynamically selects licensed frequency bands for its own use and, thus, in the process, reduces the interference from and to the users, where the channel state occupancy of the licensed primary users was assumed to be Poisson distributed. Hence, here, HMMs were employed to predict the duration of the spectrum holes for primary users. On the contrary, the HMMs and HHMMs proposed in our smart home were not used by the cognitive radio to predict the spectrum holes; instead, these models were engaged during the processing of the acquired sensor data at the base station to determine a potentially hazardous event. The working and predicting process of the two HMMs discussed are fairly similar, as by inheritance, the HMMs first train themselves by using a huge amount of data in order to develop reliable prediction models. Our proposed HMM and HHMM processes are not utilized in the cognitive spectrum sensing; in our case, the spectrum sensing is done by a very simple energy detector algorithm, and HMMs were used during the processing of the sensor’s data to determine an event.

In this paper, we analyzed an underlay RFID network with fixed transmission power near a primary user, which is a special case and an extension of our previous work [2]. The system model defined in [2] has a single source (non-RFID), ‘L’ number of relays (non-RFID) and a single destination with a primary interferer, while our proposed system is comprised of several sources (RFIDs) with a single destination and a primary interferer. Moreover, the system in our proposal is energy efficient, as compared to the one described in [2], due to the use of RFIDs. We propose a new commutative node selection criterion based on satisfying the interference constraint, while maintaining the maximum SNR of the node link at the destination. We derive closed form expressions for the probability density function (pdf) of the total SNR at the destination. We also derive closed form expressions for the outage probability of the system. Here, we have also discussed our approach to identify the taping and other known events in order to recognize unknown events, such as a leak or seepage, when they occur and track them, so as to have an efficient system with less false alarms. This type of system implementation can be seen as a cyber physical system (CPS), where the user can also give feedback and actuate certain mechanisms to achieve efficiency. We have opted for a cyber-physical approach for an efficient feedback control system for wireless sensor networks.

The remainder of the paper is arranged as follows. In the next section, we will give an overview of our system. Section 3 describes the system model and introduces various notations used throughout. Section 4 presents the received SNR statistics and the derivations of some important pdfs required for performance analysis at the destination. In Section 5, we give the details of our proposed cyber system model. Section 6 details the event detection analysis. Section 7 gives the system’s performance analysis in terms of outage probability and a discussion on the results obtained. Section 8 describes some future directions, and finally, Section 9 concludes the paper with summarizing comments.

## 2. System Overview

**Figure 1.**
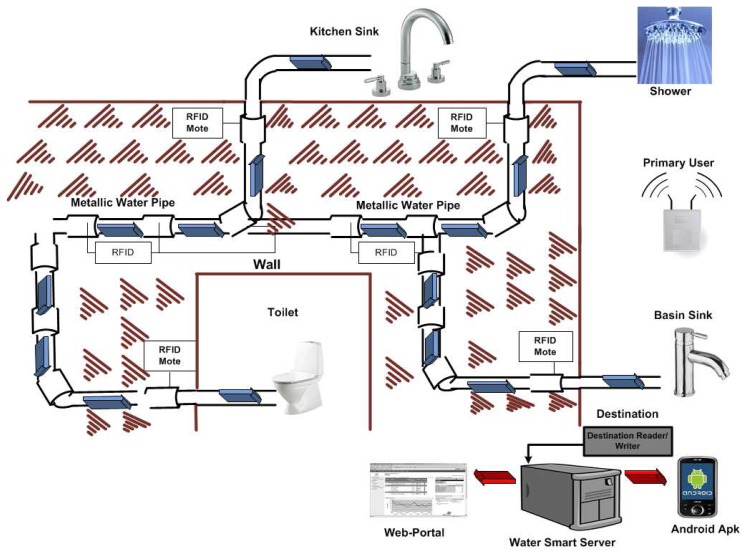
System diagram showing the RFID cognitive nodes, the primary interference and the smart home server as the destination.

In our proposed system diagram shown in Figure 1, cost effectiveness is achieved by opting for low power and low cost active RFID motes and allowing only those motes to send data that satisfy the interference threshold of the primary user. These motes can be programmed to send data after some set limit with their IDs to the reader/writer mote, and a few motes may be needed to complete the monitoring task. A simple sleep and wake-up protocol is initially utilized in the RFID motes. Once the data is received at the access mote, it first separates the data from the ID information, makes a packet and sends this packet to the water smart server through WiFi or 3G networks. At the server, the event detection and decision algorithms analyze the data and predict the future 50 or 100 states of the system. The RFID motes are renowned for their low power consumption; however, the reader/writer mote with a WiFi or 3G option will consume some energy. It will be slightly costlier to monitor the piping system than not monitoring it at all, but in the long run, it may possibly save much more by reducing maintenance and manpower costs. An Internet-based web portal application and an Android phone application running on hand held devices take the data from the server and visualize the data for the consumers. The data on the server also get archived, so that the consumers can always compare the consumption and find previous events. Due to the different nature of the events related to the timing of the occurrences, for instance the daily consumption behavior and seepage can be an hourly event, while tap opening and closing is an event that can only be detected in a few seconds’ or minutes’ time, this caused us to use hierarchical HMM, which will first look at the per second data, while checking for any unusual behavior leading to a tapping event; if no such event is detected for an hour, then the model shifts to the per-hour hierarchical level to detect consumption patterns and find the seepage. In the context of water consumption, it is expected that consumers’ behavior can change when they are regularly made aware of the amount and, in particular, the cost of what they consume.

## 3. System Model

**Figure 2.**
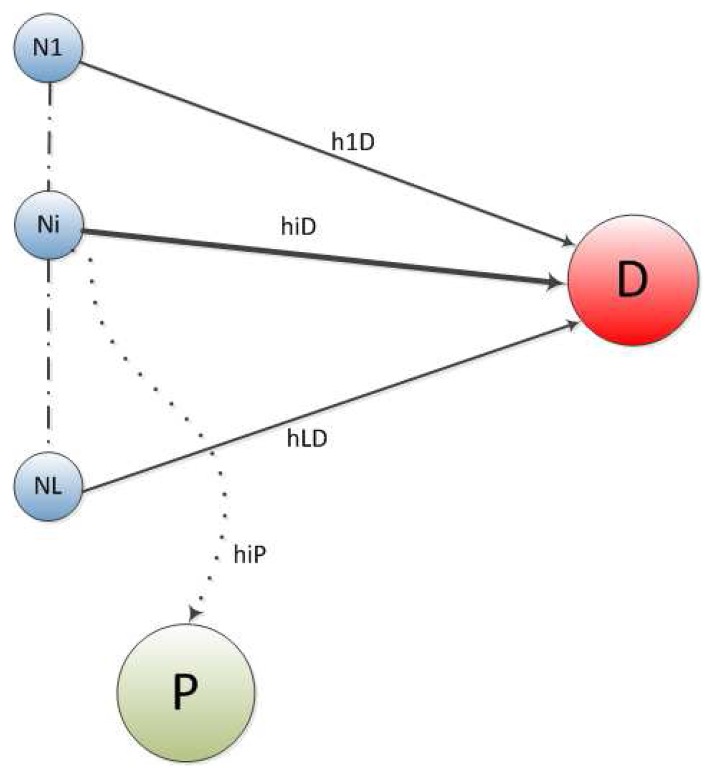
System model: RFID cognitive nodes, the destination node and the primary interferer.

We consider an underlay cognitive RFID network operating near a primary user *P*. The cognitive network consists of *L* secondary nodes broadcasting their data signals to the destination *D*, as shown in Figure 2. This broadcast is also received at the primary user *P* and causes some interference. The channel coefficients from the *i* − *th* node (*N_i_* → *D*) are *h_i_, i* = 1, 2, 3, …*L*. The interference channel from the *i* – *th* node (*N_i_* → *P*) to the primary user is *h_ip_, i* = 1, 2, 3, …*L*. In addition, we assume that the channels are subjected to additive white Gaussian noise (AWGN) with zero mean and variance *N*_0_. The available power at each node is *P*. In underlay cognitive networks, the secondary users must maintain strict interference constraint, *i.e.*, the interference at the primary user must be below a certain threshold, say *λ*. Depending upon the channel gain *h_ip_* from the *i* – *th* node to the primary user, some nodes may not be able to satisfy the interference constraint and, hence, would refrain from sending the data to the destination. We assume that the nodes estimate their interference channel when the primary user is transmitting or acknowledging any received information. This information may also be available on a dedicated feedback channel from the primary user to the *i* – *th* node in the form of a yes or no decision. Let us say *l* nodes satisfy the interference constraint out of the available *L* nodes. We group the node indexes into different sets, such as *U*, representing the set of all node indexes and *A* ⊆ *U*, the set of *l* node indexes satisfying interference constrain. We assume that all of the channel coefficients are Rayleigh distributed, and hence, their squared amplitudes are exponentially distributed.

The received SNR from the *i* – *th* node transmission can be given as:

(1) $$\gamma_{i}=\frac{P{\left|h_{i}\right|}^{2}}{N_{0}}$$

Similarly, the interference for the primary user due to the *i* – *th* node is given by:

(2) $$I_{ip}=P{\left|h_{ip}\right|}^{2}\leq \lambda$$

## 4. Received SNR Statistics

Based on the above discussion, the SNR received at the destination can be defined as the sum of the SNRs of all of the nodes satisfying the interference constraint. Mathematically,

(3) $$\begin{array}{ccc} \gamma_{T}=\underset{i∊A}{\text{∑}}\gamma_{i} & \text{such that} & I_{ip} \end{array}\leq \lambda$$

Since *I_ip_* is an exponentially distributed RV, the probability of satisfying the interference threshold is:

(4) $$P_{\lambda }=1-e^{-\frac{\lambda }{\sigma }}$$

where *σ* are the average strengths of the interfering channels. We have assumed that the nodes are present in the form of a cluster and are roughly at the same distance from the primary user. In this case, the average strengths of the interfering channels can be assumed to be the same; however, their instantaneous values may be different. Therefore, *σ*_1_*_P_* = *σ*_2_*_P_* = …. = *σ*.

Similarly, *γ_i_* are also exponentially distributed with pdf $p_{\gamma_{i}}(\gamma )=\frac{1}{\gamma_{i}}e^{-\frac{\gamma }{{\bar{\gamma }}_{i}}}$ and cumulative distribution function (cdf) $P_{\gamma_{i}}(\gamma )=1-e^{-\frac{\gamma }{{\bar{\gamma }}_{i}}}$, where *γ_i_* is the average SNR of the *i* – *th* node branch.

It is important to note that the number of nodes satisfying the interference constraint, *i.e., l* may vary from zero to *L*. If *l* = 0, the destination would not receive any signal from the nodes. For *l* = 1, only one node will be sending the data, hence no SNR aggregation is possible. In fact, the node SNR aggregation begins with *l* = 2, but the events with *l* < 2 should be included when evaluating the averages of various performance parameters. Hence, in summary, *l* nodes out of a total *L* can satisfy the interference constraint *λ* with a probability *P_λ_*. This dictates Bernoulli’s distribution; however, in order to average over all possible values of *l*, a binomial distribution should be used, which is given below:

(5) $$p_{l}(l;L;P_{\lambda })=\left(\begin{array}{c} L\\ l \end{array}\right)P_{\lambda }{\left(1-P_{\lambda }\right)}^{L-l}$$

where $\left(\begin{array}{c} L\\ l \end{array}\right)=\frac{L!}{l!(L-1)!}$.

The conditional pdf of the sum of SNR among the *l* nodes can be obtained by the following expressions.

(6) $$\begin{array}{cc} p_{\gamma_{T}(\gamma |l)}=\frac{1}{{\bar{\gamma }}_{i}}e^{-\frac{\gamma }{{\bar{\gamma }}_{i}}}; & l=1 \end{array}$$

(7) $$p_{\gamma_{T}(\gamma |l)}=\left[\underset{i∊A}{\text{∏}}\frac{1}{\gamma_{i}}\right]\underset{j∊A}{\text{∑}}\left(\frac{e^{-\frac{\gamma }{{\bar{\gamma }}_{j}}}}{\underset{k\neq j}{{\text{∏}}_{k∊A}}\left(\frac{1}{\bar{\gamma_{k}}}-\frac{1}{\bar{\gamma_{j}}}\right)}\right);l\geq 2$$

The unconditional pdf of the SNR at the destination through the node aggregation can be obtained by averaging the conditional pdf over the pdf of *l*, given as:

(8) $$p_{\gamma_{T}(\gamma )}=\overset{L}{\underset{l=1}{\text{∑}}}\left(\begin{array}{c} L\\ l \end{array}\right)P_{\lambda }^{l}{\left(1-P_{\lambda }\right)}^{L-l}p_{\gamma_{T}(\gamma /l)}$$

Hence, substituting Equation (6) in Equation (8), we have,

(9) $$\begin{array}{c} p_{\gamma_{T}(\gamma )}=\underset{l=1}{\underset{︸}{P_{\lambda }^{1}{\left(1-P_{\lambda }\right)}^{L-1}\overset{L}{\underset{i=1}{\text{∑}}}\frac{1}{{\bar{\gamma }}_{i}}e^{-\frac{\gamma }{{\bar{\gamma }}_{i}}}+}}\\ \underset{l\geq 2}{\underset{︸}{\overset{L}{\underset{l=2}{\text{∑}}}\left(\begin{array}{c} L\\ l \end{array}\right)P_{\lambda }^{l}{\left(1-P_{\lambda }\right)}^{L-l}\left[\underset{i∊A}{\text{∏}}\frac{1}{\gamma_{i}}\right]\underset{j∊A}{\text{∑}}\left(\frac{e^{-\frac{\gamma }{{\bar{\gamma }}_{j}}}}{\underset{k\neq j}{{\text{∏}}_{k∊A}}\left(\frac{1}{{\bar{\gamma }}_{k}}-\frac{1}{{\bar{\gamma }}_{j}}\right)}\right)}} \end{array}$$

The cdf of *γ_t_* can be obtained by integrating Equation (9) from zero to ∞ given below:

(10) $$\begin{array}{c} P_{\gamma_{T}(\gamma )}=\underset{l=1}{\underset{︸}{P_{\lambda }^{1}{\left(1-P_{\lambda }\right)}^{L-1}\overset{L}{\underset{i=1}{\text{∑}}}(1-e^{-\frac{\gamma }{{\bar{\gamma }}_{i}}})+}}\\ \underset{l\geq 2}{\underset{︸}{\overset{L}{\underset{l=2}{\text{∑}}}\left(\begin{array}{c} L\\ l \end{array}\right)P_{\lambda }^{l}{\left(1-P_{\lambda }\right)}^{L-l}\left[\underset{i∊A}{\text{∏}}\frac{1}{\gamma_{i}}\right]\underset{j∊A}{\text{∑}}\left(\frac{{\bar{\gamma }}_{i}(1-e^{-\frac{\gamma }{{\bar{\gamma }}_{j}}})}{\underset{k\neq j}{{\text{∏}}_{k∊A}}\left(\frac{1}{{\bar{\gamma }}_{k}}-\frac{1}{{\bar{\gamma }}_{j}}\right)}\right)}} \end{array}$$

## 5. Performance Analysis

In this section, we derived closed form expressions for the outage probability of the system using the results obtained in the previous section.

### 5.1. Outage Probability

A communication system is said to be in an outage when the received SNR is fallen below a certain threshold *η*. The total SNR expression in Equation (9) is for the situation when at least one node satisfies the interference constraint. However, as mentioned earlier, it is possible that none of the relays satisfy the interference constraint. The probability of this event is (1 − *P_λ_*)*^L^*. Hence, the outage probability of the system can be evaluated as:

(11) $$P_{out}=P_{\gamma_{T}}(\eta )+{\left(1-P_{\lambda }\right)}^{L}$$

## 6. Numerical Results

In this section, we present simulation results to verify the derived analytical expressions. First, we define the parametric setup for the simulations, and later, the results are discussed in detail.

### 6.1. Simulation Setup

All of the simulation results are generated by varying the average SNR *γ_i_*, where $\gamma_{i}=\alpha \_i\frac{P}{N_{0}}$. The value of *α* = 0.1 in the case of an interference channel. The noise is considered to be AWGN with zero mean and unit variance. The transmission power at the nodes is also assumed to be *P_N_* = 1. The maximum number of nodes in the system is *L* = 5, and all of the situations are compared with equal power conditions.

### 6.2. Discussion

**Figure 3.**
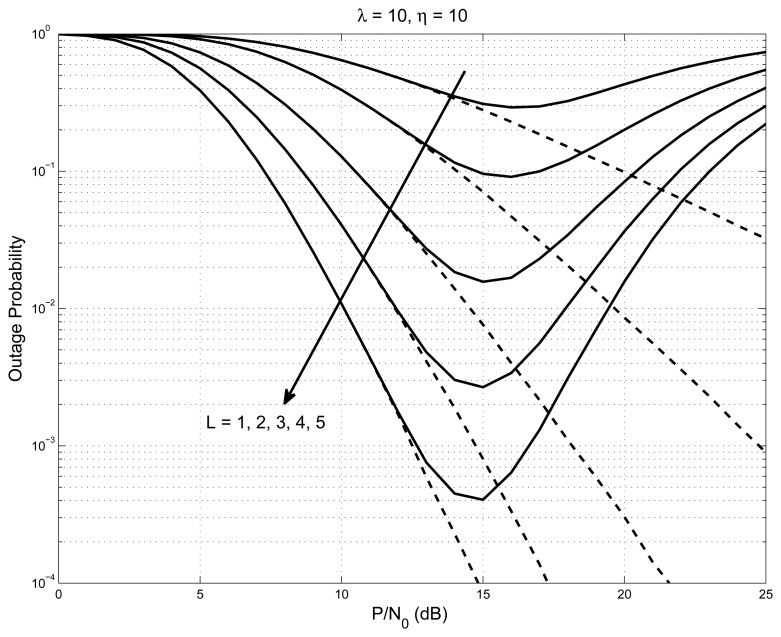
Outage probability of the system with *L* = 1, 2, 3, 4, 5 for interference constraint *λ* = 10 and outage threshold *η* = 10.

**Figure 4.**
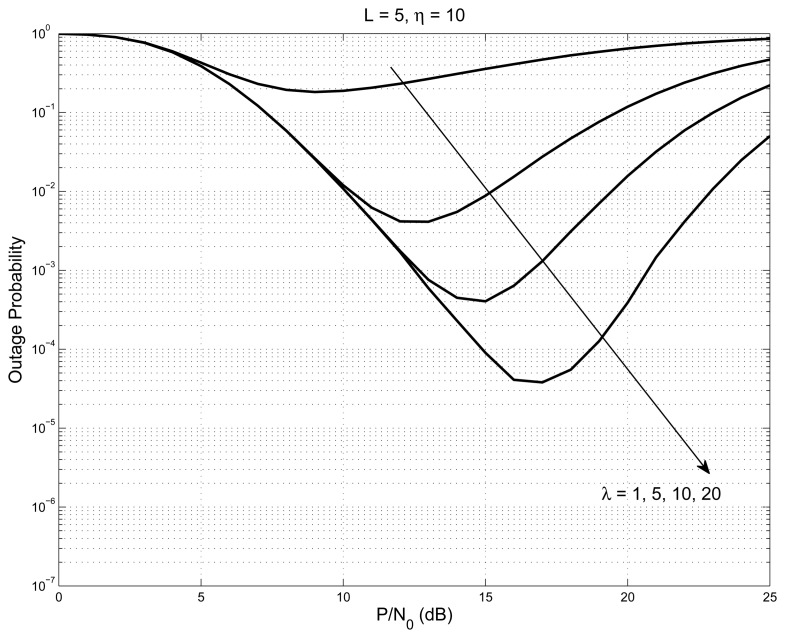
Outage probability of the system with *L* = 5 for interference constraint *λ* = 1, 5, 10, 20 and outage threshold *η* = 10.

The outage performance of the system is plotted in Figure 3 with *L* = 1, 2, 3, 4, 5, and the values of interference constraint and outage threshold are set to be 10. The outage graph initially follows the regular water-fall curve, until it reaches a point where the nodes started to have better SNR than the outage threshold. At this point, the outage graph shows an increase of probability. As the number of nodes increases, the outage graph’s probability values decrease steeply (shown as a solid line). On the same graph, we have shown the case of the interference constraint having a very large value (shown as a dashed line); all of the nodes satisfy the interference constraint all of the time, and hence, the system acts like a non-cognitive network. The graph shown in Figure 4, is with *L* = 5 and outage threshold = 10 with varying interference constraint *λ* = 1, 5, 10, 20. The figure shows that the elbow point, indicating the event when the nodes started to meet the interference constraint, slopes down with the increase in the interference constraint.

**Figure 5.**
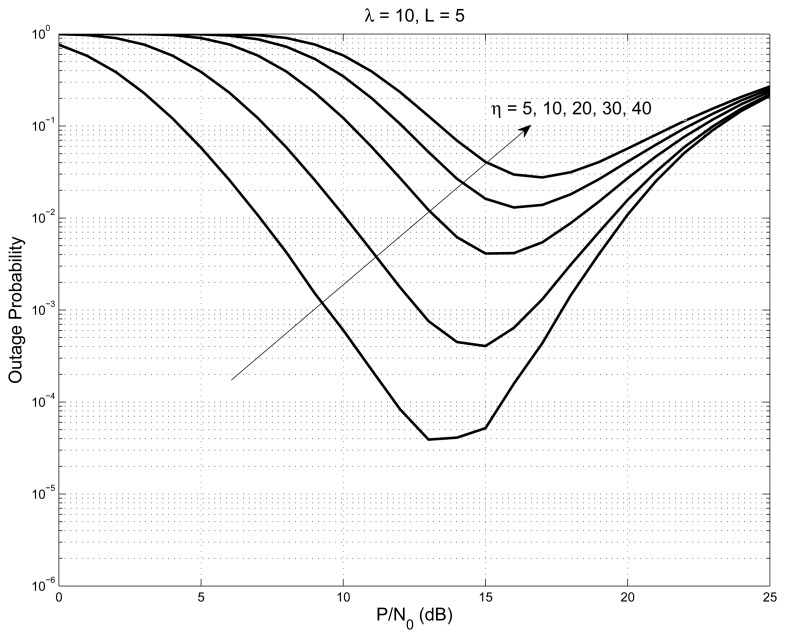
Outage probability of the system with *L* = 5 for interference constraint *λ* = 10 and outage threshold *η* = 5, 10, 20, 30, 40.

Figure 5, shows the outage probability when *L* = 5, and the interference constraint is set to *λ* = 10 and varying the values of the outage threshold *η* = 5, 10, 20, 30, 40. It shows that the probability decreases with the increase in the outage threshold.

## 7. Proposed Model for Cyber System

**Figure 6.**
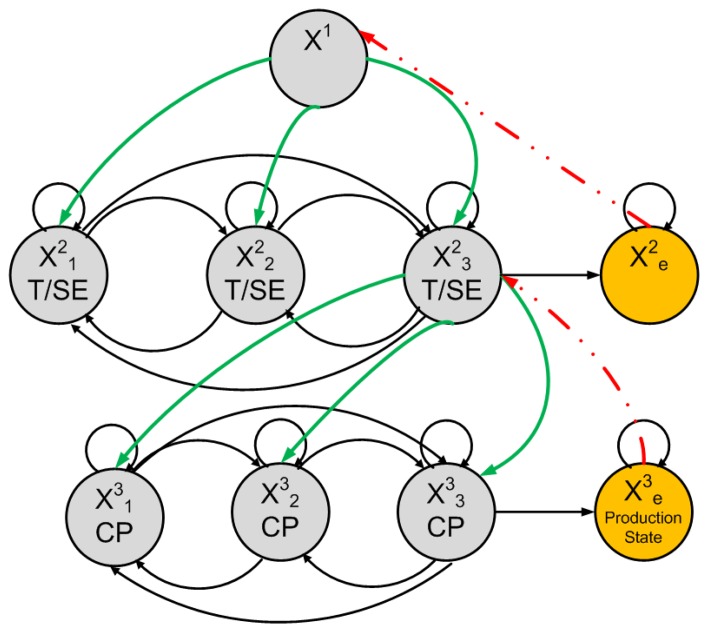
Proposed HHMM for event detection and future event prediction. (CP, consumption pattern event; T/SE, tap and seepage event.)

Our smart home system diagram is shown in Figure 1. Our proposed model will allow us to detect, as well as to predict various events of interest. Every state is associated with a probability distribution over the possible output symbols. In our context, an event can be a tap event, high/low usage of water and/or seepage. The HHMM to solve and predict the tap, seepages and consumption pattern events is shown in Figure 6. An off-the-shelf 125-KHz RFID module was utilized in the experiments. Pressure sensors were deployed at the main tapping points to emulate the taping and seeping events. However, due to the requirement of a large data set in order to predict the events correctly, pseudo pressure data closely matching the actual received data was constructed to get the predictions.

### 7.1. Hierarchical Hidden Markov Models

Hierarchical hidden Markov models [7] are simplifications of HMMs, which provide an answer to two main problems that arise in complex sequence modeling. At first, HHMMs can correlate events that happen comparatively distant from each other in an observation sequence and still maintain the ease and flexibility of a simple Markov process. Hierarchical hidden Markov models (HHMMs) are structured multi-level stochastic processes. HHMMs make each of the hidden states an autonomous probabilistic model, hence making each state an HMM, as well. Therefore, the states of an HHMM emit sequences rather than a single symbol. Figure 6 shows our HHMM to determine consumption patterns, tap events and to predict seepage events hierarchically; the third state is also an HMM to detect the usage patterns on top of seepage and tap events. An HHMM works by generating sequences repeatedly through activating one of the lower states of a selected state, since each HHMM is generally made up of a standard single-level HMM. Therefore, the individual states of the HMM are, in fact, the production states of the next HHMM, having a non-zero probability of going from any one state to another state. This process of recursive activations ends when we reach a special state, called the production state. In our context, the production state is the final state of the third-tier hierarchy of consumption patterns. The output symbols are only emitted through the production states; this output symbol is picked out from the set of output symbols according to a probability distribution, while the internal states are hidden states that do not give off observable symbols directly. The control then returns to the state that initiated the algorithmic activation chain. This constitutes a tree structure, where the node at the top of the structure is called the root state, while the production states makes the leaves.

## 8. Event Detection and Analysis

**Figure 7.**
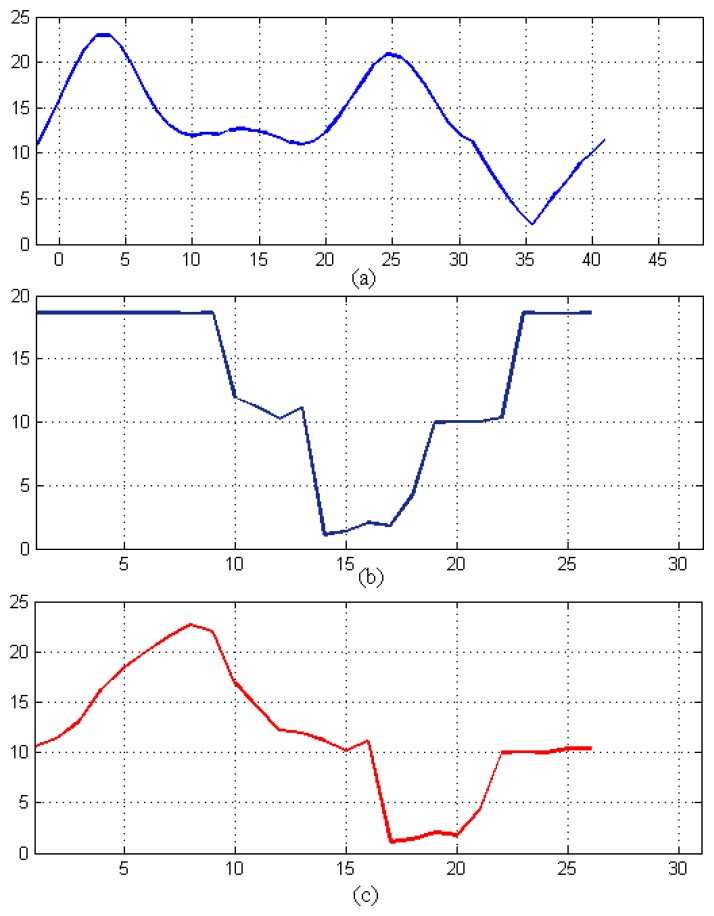
Pseudo pressure values in ‘bar’ on the y-axis for (**a**) consumption pattern (CP) (per hour on the x-axis), (**b**) tap event (TE) (per second on the x-axis) and (**c**) seepage event (SE) (per second on the x-axis).

To determine patterns, a model has to be formulated that can be utilized to identify real instances of abnormal events from the suspicious ones. Because of this, the model should detect possible abnormal activities, expeditiously examine large sets of data and produce hypotheses with only partial and fallible information. The transition-based model shown in Figure 6 identifies the event by comparing the anticipated outputs with the data sets from different nodes in the network. It is used to detect the water consumption patterns, seepage and tap events hierarchically. We have used the expectation-maximization (EM) algorithm for HMM parameter training and performed the hypothesis testing using the maximum likelihood (ML) principles from [1,4] to identify the data samples as either normal, a possible event or a confirmed event during the recognition phase of the algorithm; as the stability is directly proportional to the amount of data collected and the number of events occurring. Our proposed system will periodically train itself, and as the data set grows, the stability increases. the initial transition matrix when there were no real data sets available will be stationary, while we next obtain the matrix of transition and emission probabilities. Here, we estimate these probabilities based on our pseudo data. The emission matrix is initialized based on the following assumptions; if usage increases, then there is a 30% chance of the need to repair and expand the water supply network. If seepage and leak events increase, then there is a 50% chance that we need to repair the system. If the water utility cost increases, then there is a 20% chance that we need to conserve water. We have logically assumed the emission matrix with a 30% chance for the water system expansion and a 50% chance for repair. This matrix can be determined through real analysis of the home piping system during one year. In short, we can set the emission matrix values on the analyzed real data, but here, for simplicity, we assumed the matrix. Furthermore, we can map the location of the seepages and can alert households to take evasive actions. After collecting a sufficiently large data set, large enough to train our model to give meaningful predictions and decisions, we can tune the model parameters, such as the transition, emission and initial probabilities, so as to maximize the system’s event detection performance. For instance, a data set of a mere few hours is sufficient for the detection of a taping event, while a consumption event will require at least the data of the whole day. Given this model, we use the forward HMM algorithm to generate predicted states for the three features of interest illustrated in Figure 7. The predictions were made after adjusting the transition and emission probabilities over 500 iterations.

**Figure 8.**
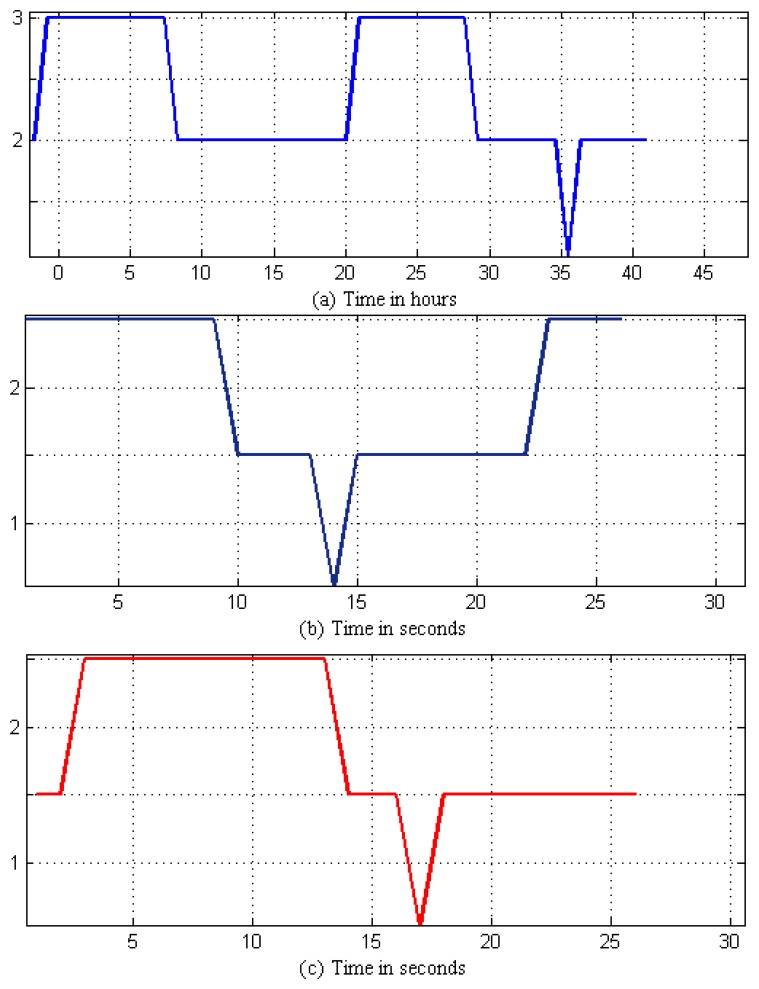
(**a**) Consumption patterns (CPs) (per hour on the x-axis); (**b**) tap event (TE) (per second on the x-axis); and (**c**) seepage event (SE) (per second on the x-axis); quantized state sequences are on the y-axis.

**Figure 9.**
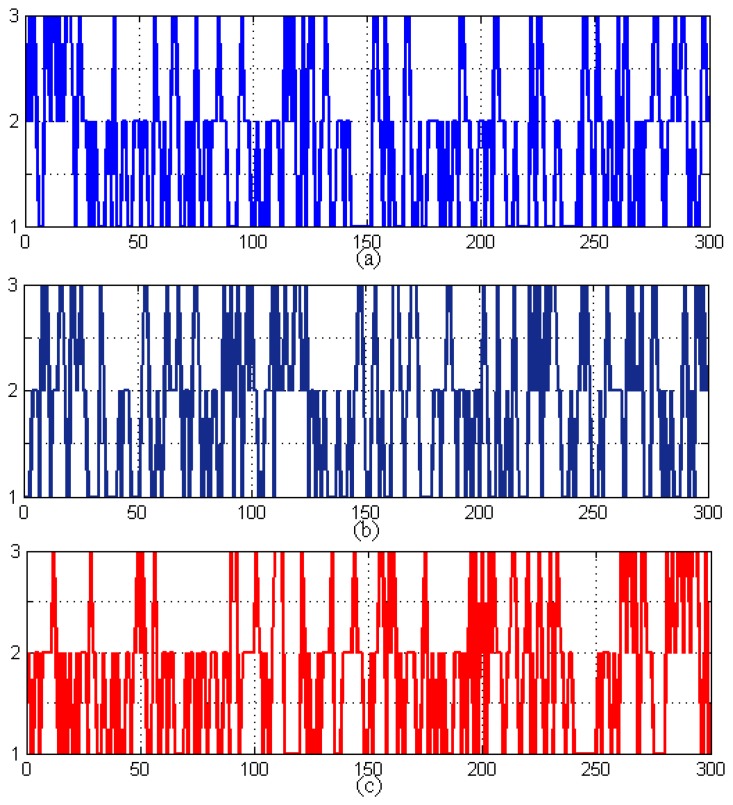
(**a**) Consumption pattern (CP) predicted states; (**b**) tap event (TE) predicted states and (**c**) seepage event (SE) predicted states against 300 future points (hours for (**a**) and seconds for (**b**) and (**c**)) from HHMM.

Tap events occur within the consumption pattern measurements; this makes it easier and efficient to model using a single HHMM rather than two HMMs. HHMMs train themselves after getting hourly, daily and monthly data and determining the events from this data set, so it may take as short as a couple of hours to capture the tapping events and a minimum of two days to determine the daily consumption patterns. Figure 7 shows the pseudo daily consumption pattern and tap event pressure data. These data are quantized to determine the systems’ observation sequence states in Figure 8. The HHMM starts with the root state, which can, in turn, activate and pass the control to any one of the internal states at the second hierarchy, according to an initial probability matrix. The third sub-state of the second hierarchy of the consumption measurements can also pass the control to the tap event states at the third hierarchy, according to its own initial non-zero probability matrix. The third tier predicts the tapping event, generates the outcome and passes the control to the hierarchy that activated it through the production state. After that, the second hierarchy completes its generation of the predicted events and passes the control back to the root state. As the data set grows and the system comes across a number of events, our proposed HHMM trains itself using daily and monthly event data sets. This training and parameter determination can be done in a few minutes. Figure 9 shows the generated state prediction for the two events. HHMM generated prediction states indicate what may be the expected behavior of the system. Given the current obtained readings, the higher the prediction span, the more complex will be the decision. Therefore, we need an algorithm to detect these predictions to come up with any sort of decision. These HHMM-based predictions for 300 points of data from each node are given to a decision algorithm, which searches for 50 or more continuous State 3 predictions in the predicted sequence for each node and compares them with the other nodes in the region. If three or more nodes show similar predicted behavior, then a SMS alert is sent to the households and an alarm signal to the water smart server. The process then waits for acknowledgment, and after receiving it, the system provides visualization of the event with the location of the event. These 300 points indicate hours in the case of the consumption pattern and seconds in the case of tap and seepage events. In other words, we can make HHMM predict a sequence of any number of points (hours, seconds) regarding the consumption, tap and seepage events. The output of Figure 9 shows that the predicted events hop around in three distinct states. If the predicted pattern depicts more transactions in State 3, this means that we have more chances of having an actual event in the future, and if the predicted sequence mostly remains in the second or first states, the chances of having this event are very rare.

## 9. Future Work

Human-centric sensing allows us to derive the most out of water monitoring research and applications. We intend to enhance our system with a multi-interface data service for administrative functions and a map service for normal users who are interested in the overall consumptions and ways to conserve water. It can also utilize existing sensor modules and feed their data to the users’ hand-held computing devices for processing and analysis. We are exploring the use of communication methods, such as WiFi, Bluetooth, *etc.* These provide a wide choice of data transfer speeds and flexibility in building a network. At the later stage of the research, an improved energy-efficient event-driven MAC protocol for in-pipe RFID motes can be introduced. Some of the applications where this system can also be applied are sewage monitoring, oil and gas installations, industrial gas leak detections and quality management.

## 10. Conclusions

We proposed a best node selection scheme for a cognitive network operating near a primary user. We deduced through analysis and simulations that SNR is not the only criterion to pick up the best node in the cognitive setting. The proposed scheme works by first eliminating those nodes that do not satisfy the interference constraint. Then, among those nodes that successfully satisfy the constraint, the one giving the maximum end-to-end SNR is chosen to forward the source message to the destination. We derived the closed form pdf of the total SNR at the destination using the MGF approach and then used it to derive BER and outage probability in closed forms. Analytical formulae are verified through simulations. Some important features and tradeoffs of the proposed scheme are also discussed. Our key interest is to conserve water and control the cost by sensing various aspects of the water network in a water smart home and to share the information to users to control, preserve and improve their life styles. The end-user, which can be a household or an authority, is informed each day about the consumption and can decide how to be more efficient. This paper presents a human-centric CPS cycle for an in-pipe water monitoring system. The CPS cycle and the proposed models for consumption patterns and tap opening/closing events for the water network system have been described. We also have described the application of HHMMs for modeling the system and their use for detecting events, identifying patterns in the data, predicting events and in making decisions. We also presented modeling results and analysis based on the pseudo pressure data. In addition, directions for further research and development and its impact were presented.
